# Reactivity and regulation of negative and positive emotions in child- and adolescent diagnostic and trait-level ADHD: a cross-sectional study

**DOI:** 10.1186/s12888-025-07708-0

**Published:** 2025-12-23

**Authors:** Rebecka Astenvald, Matilda A. Frick, Johan Isaksson

**Affiliations:** 1 Department of Medical Sciences, Child and Adolescent Psychiatry Unit, Uppsala University, Uppsala, Sweden; 2 Department of Psychology, Stockholm University, Uppsala, Sweden; 3 Center of Neurodevelopmental Disorders (KIND), Centre for Psychiatry Research, Department of Women’s and Children’s Health, Karolinska Institutet & Stockholm Health Care Services, Region Stockholm, Stockholm, Sweden

**Keywords:** ADHD, Emotional reactivity, Emotion regulation, Sex, Anger, Exuberance, Comorbidity

## Abstract

**Background:**

Emotional difficulties are common in attention-deficit/hyperactivity disorder (ADHD), but previous research lack specificity regarding how reactivity and regulation of distinct emotions relate to categorical (diagnostic) and dimensional (trait-level) ADHD, while also clarifying sex- and age-related patterns. Considering co-occurring psychiatric traits is important due to the transdiagnostic nature of emotional challenges.

**Method:**

This cross-sectional study included two samples: a diagnostic cohort (*N* = 104, 10–17 years, 56.7% females) consisting of participants with an ADHD diagnosis (*n* = 56) and typically developing controls (*n* = 48), and a cohort referred to a first visit to a specialist psychiatric unit (*N* = 85, 13–18 years, 78.8% females) for which a dimensional measure of ADHD was used. Linear regressions were used to assess associations between ADHD and self- and caregiver-rated reactivity (frequency/intensity) and regulation (by oneself or with the help of others) of four different emotions (sadness, fear, anger and exuberance) using the Emotion Questionnaire. Effects of sex were explored. Adjustments were made for age and co-occurring psychiatric traits (anxiety, depression, conduct problems and autism) within the referred cohort. Findings were validated through caregiver ratings for the diagnostic cohort (N=103) and the referred cohort (N=212).

**Results:**

Diagnostic and trait-level ADHD were robustly linked to reactivity and a limited ability to regulate (i.e., dysregulation) anger. Diagnostic ADHD was also related to dysregulation of exuberance and fear, and higher levels of sadness and fear reactivity. Trait-level ADHD was, in addition to anger, associated with elevated reactivity and dysregulation of exuberance, adjusting for co-occurring psychiatric symptoms. No interaction effects of sex by ADHD were identified.

**Conclusion:**

Anger may be particularly relevant for ADHD. Reactivity and dysregulation of exuberance may also be important for ADHD, although further research is needed to clarify the relation between ADHD and positive emotions. Potential effects of sex and co-occurring psychiatric traits are discussed.

**Supplementary Information:**

The online version contains supplementary material available at 10.1186/s12888-025-07708-0.

## Introduction

Attention-deficit/hyperactivity disorder (ADHD) is a neurodevelopmental condition where symptoms are present in multiple settings and result in functional impairment [1]. Although ADHD is defined by the core symptom domains of inattention and/or hyperactivity/impulsivity, associated features include emotional difficulties [1]. Elevated *emotional reactivity* (i.e., responses to emotional evoking stimuli, including changes in subjective feelings, behaviour and physiology) and *emotion dysregulation* (i.e., difficulties in identifying or balancing emotional responses, adhering to emotion regulation goals and using adaptive regulation skills) [2] are reported in approximately 25–50% of children with ADHD and often result in a more adverse clinical prognosis [3, 4].

Previous research has often used a broad measure of emotion dysregulation encompassing several emotional factors, possibly resulting in a lack of specificity. It is important to distinguish between reactive and regulatory components of experiencing and handling emotions as these are conceptually distinct from each other, only modestly correlated, and may be differentially related to behavioral and functional outcomes, including peer problems [5–7]. Further, it is important to acknowledge different emotions, since reactivity and dysregulation of both *negative* and *positive* emotions may be characteristic of ADHD [3, 8]. Increased specificity is also essential since emotional difficulties are not unique to ADHD, but play a significant role in psychopathology overall [e.g., 9–13]. Accordingly, both emotion reactivity and dysregulation are recognized as transdiagnostic factors [14–16], and are as such included in the Research Domain Criteria framework [17]. Disentangling condition-specific emotional patterns could lead to a better characterization of how emotional reactivity and dysregulation relate to ADHD and a more precise nosology [18]. Furthermore, according to the affective neuroscience framework, emotions are understood as arising from distinct affective systems with specific neurobiological underpinnings [19]. These systems are both theoretically and empirically linked to various behavioural patterns, including those that characterize the core symptoms of ADHD [20].

In order to differentiate between emotional factors, Rydell and colleagues [5] proposed a framework including *reactivity* and *regulation* of negative (i.e., fear, sadness and anger) and positive (i.e., exuberance) emotions. Through their framework, reactivity and dysregulation of anger, as well as fear and sadness reactivity, have been linked to externalizing problems (including ADHD symptoms) during childhood [5, 7, 21, 22]. However, these studies are based on non-clinical populations and may not generalize to a clinical context. Sjöwall and colleagues [23] found that a clinical diagnosis of ADHD in children aged 7–13 years was associated with increased *dysregulation* across all emotions compared to typically developed controls. The authors, however, did not investigate emotional reactivity nor included older adolescents. The latter may be of particular importance given the suggested increase in negative emotions throughout adolescence [24] and since adolescence is a period characterized by numerous developmental changes [25]. Further, emotion regulation ability may improve with age, but can also decrease during specific periods of adolescence associated with higher intensity of negative emotions [26]. Carta and colleagues [27] found that children and adolescents with diagnostic ADHD had higher levels of negative emotionality than the comparison group, albeit the emotionality construct used did not distinguish between specific emotions. This is an important omission as other studies suggests that specifically anger or irritability may be relevant for ADHD [e.g., 28–30], albeit research on sadness and fear in clinical populations is scarce, making it unclear whether all negative emotions, or only specific ones, are related to ADHD.

On a dimensional level, studies including clinical samples have been more equivocal, with both reports of a significant association [31] and no significant association [27] between negative emotional reactivity and ADHD symptoms. These mixed findings elucidate the importance of examining both diagnostic (categorical) and trait-level (dimensional) ADHD, as the associations with emotion-specific reactivity and regulation may differ depending on the degree of symptoms, where diagnostic ADHD represent the extreme end of a continuum of ADHD traits [32].

Positive emotions may also play a specific role in ADHD [e.g., 23, 30, 33, 34]. In particular, *surgency* (a temperamental construct closely related to exuberance), defined by positive emotional reactivity, pleasure/sensation-seeking and high activity level, has been emphasized as an important emotional domain in ADHD [28, 30, 35]. Notably, the relevance of surgency for ADHD may not be explained solely by the conceptual overlap with ADHD [34]. In non-clinical populations, reactivity and dysregulation of exuberance have been associated with externalizing problems, including ADHD symptoms, in young children [5, 7, 21]. Corroborating this, surgency have been linked to increased externalizing difficulties, as well as ADHD symptom severity, among children with elevated ADHD symptoms [36]. Conversely, other studies have found no difference regarding surgency for individuals with ADHD compared to typically developed controls [27] or relation between surgency and ADHD in a clinical sample [37]. These findings necessitate further research as positive emotion dysregulation has been suggested to play an important role in future psychiatric difficulties and impairment [8].

Due to high comorbidity rates in ADHD and the transdiagnostic characteristics of emotional reactivity and regulation, co-occurring psychiatric symptoms are important to consider. Okado and colleagues [34] found that individuals with ADHD and co-occurring internalizing conditions had higher levels of negative emotional reactivity than individuals with ADHD and co-occurring externalizing conditions, indicating that the relation between negative emotionality and ADHD may be explained by internalizing symptoms. However, this effect may not apply for all negative emotions, as for example irritability share genetic factors with ADHD [33]. Other common co-occurring conditions include autism [38], which is often overlooked despite its association with emotion regulation difficulties [39], as well as conduct disorder [40], where the impact of conduct problems on the associations between ADHD and emotional difficulties remain unclear [23, 41].

Moreover, females with ADHD may experience more emotional difficulties than males with ADHD [42, 43]. This may be particularly pronounced during adolescence, when females typically experience higher rates of depression and some anxiety conditions compared to males [44]. Specifically, girls with ADHD may have higher levels of negative, but not positive, emotional reactivity than boys with ADHD [34]. However, others have found no sex differences regarding emotional difficulties in diagnostic ADHD [23, 45, 46], why more research is needed.

In sum, there is a need to clarify the role of specific emotions and regulation thereof in ADHD within a clinical context, also considering potential effects of sex and co-occurring psychiatric traits, since emotional difficulties significantly contributes to the clinical burden. Further, by examining specific emotion domains, we target distinct affective systems [19] that could help us better explain individual differences in ADHD presentations, inform treatment personalization and expand beyond purely cognitive models of the condition. The aim of the present study was to examine how reactivity and regulation of positive and negative emotions relate to diagnostic and trait-level ADHD. First, we investigated emotional reactivity and regulation for different emotions in children and adolescents with diagnostic ADHD compared to typically developed controls, exploring sex effects and adjusting for age. We hypothesized that diagnostic ADHD would be associated with higher reactivity and dysregulation across all emotions. Second, in a group of adolescents referred to a specialist psychiatric unit, we examined the associations between trait-level ADHD and reactivity and dysregulation across all emotions, exploring sex effects while adjusting for age and other psychiatric symptoms. We hypothesized that ADHD symptoms would be specifically related to reactivity and dysregulation of anger and exuberance. Due to the mixed findings and scarce research regarding reactivity and regulation of fear and sadness, as well as sex-related effects, we adopted an exploratory approach for these analyses.

## Method

### Participants and procedure

#### The diagnostic cohort

Participants with ADHD were recruited from the child and adolescent psychiatric (CAP) outpatient unit in Uppsala, Sweden, via telephone, post and/or flyers. Inclusion criteria consisted of a clinical diagnosis of ADHD and being 10–18 years old. Exclusion criteria included autism, intellectual disability, psychosis and long-acting medication for ADHD. Data were collected during June 2021 – September 2023. Two participants were excluded due to reporting a diagnosis of autism and two participants were excluded due to incomplete consent forms. Based on caregiver ratings, 42.6% of the participants had ADHD-medication, although medication data for two participants were missing.

A comparison group of typically developing controls were recruited via post through the Population Register in Uppsala and Meta (i.e., Facebook). Exclusion criteria included having a psychiatric diagnosis, as reported by caregivers. One participant was moved from the comparison group to the ADHD group due to 1) screening positively for ADHD on the ADHD module of the Mini-International Neuropsychiatric Interview for Children and Adolescents (MINI-Kid) during study participation [47] and 2) fulfilling six or more criteria for ADHD on the parent-rated ADHD Self-Report Scale for adolescents (ASRS-P) [48]. Five participants were excluded due to scoring above cut-off for ADHD on MINI-Kid, but where data on functional impairment related to ADHD symptoms were unavailable, hindering further assessment of diagnostic status.

A total of 104 children and adolescents (*n* = 56 with ADHD and *n* = 48 typically developing controls) were included in the final analyses. Findings were validated via caregiver ratings, where 55 reports (72.7% mothers) were included for the diagnostic cohort and 48 reports (83.3% mothers) were included for the comparison group, with participants largely overlapping with the adolescent ratings. Sample characteristics are presented in Table 1.

**Table 1:** Sample characteristics

	Diagnostic cohort					
	ADHD		Comparison group		Referred cohort	
	Self-reports n = 56	Caregiver reports n = 55	Self-reports n = 48	Caregiver reports n = 48	Self-reports N = 85	Caregiver reports N = 212
Age, m years (range)	13.48 (10–17)	13.49 (10–17)	13.56 (10–17)	13.85 (10–17)	15.71 (13–18)	13.17 (6–18)
Sex, % females	64.3%	63.6%	47.9%	45.8%	78.8%	62.3%
Highest educational level^a^ for parents, %	66.7%	67.3%	90.7%	91.7%	77.4%	
Occupational status^b^ for parents, %	96.3%	96.4%	98.9%	99.0%	98.8%	
	ADHD		Comparison group			
ADHD diagnosis						
Combined, n	29		0			
Predominantly inattentive, n	18		0			
Predominantly hyperactive/impulsive, n	5		0			
Unspecified, n	1		0			
MINI-Kid ADHD criteria^c^, m (SD)	12.69 (3.76)		2.74 (2.89)			

*Note*: Sex = assigned sex at birth, ADHD: attention-deficit/hyperactivity disorder. MINI-Kid: Mini-International Neuropsychiatric Interview for Children and Adolescents

^a^ The percentage of cases where at least one caregiver has a university-level education, based on caregiver ratings

^b^ The proportion of caregivers having an occupation (e.g., currently employed or studying), based on caregiver ratings

^c^ The average number of fulfilled MINI-Kid criteria based on the ADHD module. Note that two participants in the ADHD group, and one participant in the comparisongroup, had missing data

#### The referred cohort

All patients aged 13–18 years referred to the CAP unit in Uppsala for psychiatric difficulties, as well as their caregivers, complete the Electronic Psychiatric Intake Questionnaire (EPIQ), including several questionnaires targeting psychiatric symptoms, as part of a screening process. Patients and caregivers who choose to participate in research completed an additional questionnaire, namely the Emotion Questionnaire (EQ) [5]. Upon agreeing to participate in research, researchers are granted permission to access their answers on the questionnaires included in EPIQ. Data were collected during December 2022 – December 2023. Duplicate ratings were found for adolescents (*n* = 1) and caregivers (*n* = 10). Duplicates were excluded by the following criteria: 1) Complete data were prioritized over non-complete data, 2) ratings from the caregiver who resided with the child was prioritized, 3) a random selection for one of the ratings was made if the above-mentioned criteria could not be applied. If one informant had two submissions, the most recent rating was chosen. A total of *N* = 85 adolescents were included in the final analyses. Caregiver reports (*N* = 212) were used to validate findings, which included additional informants as more caregivers than adolescents completed the EPIQ, also including a broader age range. Sample characteristics are presented in Table 1.

Participation in the study included filling out self- and caregiver-rated questionnaires on a digital platform. The study was pre-registered at the Open Science Framework (https://osf.io/uj4xg).

Occupational status and educational level was unavailable for seven participants in the adolescent diagnostic cohort due to missing caregiver-rated data. For the referred cohort, missing data was found for educational level (*n* = 1). Frequencies of type of ADHD diagnoses are based on caregiver reports, where missing data was found for two individuals with ADHD and five typically developing participants. MINI-Kid interviews during the study were held either with the participant and their caregiver/s (39.4%) or with the participant alone (41.3%). Informant data were missing for 17.3%. Time constraints hindering assessments during the study led to using previous MINI-Kid results from the CAP unit’s medical records for two participants with ADHD (1.9%).

## Measures

### Emotional reactivity and regulation

Emotional reactivity and regulation were measured by self- and caregiver ratings of the EQ [5, 19]. The short version of the EQ consists of 16 items, where two items target *emotional reactivity* for each emotion (i.e. fear, sadness, anger and exuberance [e.g., “often gets angry” and “react/s strongly when angry”]), and two items target *emotion regulation* for each emotion (e.g., “when angry, he/she can make themselves calm” and “when angry, it is easy for others to make her/him calm”) [5]. Items are rated on a scale ranging from 1 (doesn’t apply at all) to 5 (applies very well). Higher scores on the reactivity items indicates elevated emotional reactivity, whereas higher scores on the regulation items represents better regulation capacity. Overall, the EQ has shown high test-retest reliability, as well as acceptable convergent validity [5]. As a measure of internal consistency, for each emotional construct, items were moderately to largely correlated, with inter-item correlations ranging from *r* = 0.37 to 0.74. See Supplementary Table S1 for details. The means of reactivity and regulation for each emotion were used in the analyses, with self-ratings used for the main analyses and caregiver ratings as a validation set.

### ADHD

For individuals with ADHD in the diagnostic cohort, diagnostic status was assessed a priori by the CAP unit. Assessments typically included a minimum of two visits, in which the clinician took medical and developmental history, and administered the MINI-Kid [49]. The clinician determined diagnostic status in accordance with the Diagnostic and Statistical Manual of Mental Disorders 5^th^ edition [1]. Based on caregiver reports, the mean time between receiving a diagnosis from the CAP unit and participating in the study was 1.1 years. Diagnostic status was further assessed via the ADHD module of MINI-KID for the diagnostic cohort during study participation to inquire about current symptomatology and as a screening procedure for the comparison group.

For both cohorts, ADHD symptoms were measured through self-ratings of the ASRS (ASRS-A) [50] and caregiver ratings of ASRS-P [48]. Each scale consists of 18 items corresponding to the core symptom domains of ADHD. Items are scored on a scale ranging from 0 (never) to 4 (very often), where higher scores indicate more ADHD symptoms. Both scales have been validated in adolescent samples with good concurrent validity [48, 50]. The mean of each scale was used as a measure of ADHD symptoms. Cronbach’s alpha was α = 0.95 (children and adolescents) and α = 0.97 (caregivers) for the diagnostic cohort, and was α = 0.92 (adolescents) and α = 0.94 (caregivers) for the referred cohort.

### Co-occurring psychiatric symptoms

Measures of co-occurring psychiatric symptoms were included for the referred cohort only. This was because only a subsample of the diagnostic cohort, participants aged 13–18 years, completed questionnaires on co-occurring psychiatric symptoms. Thus, there was limited statistical power in the diagnostic cohort to examine these symptoms.

Anxiety was assessed by the Spence Children’s Anxiety Scale for caregivers (SCAS-P) [51] á 38 items, and its short version for adolescents (SCAS-S) [52] á 19 items. Items are rated on a scale ranging from 0 (never) to 3 (always), where higher scores indicate more symptoms of anxiety. Both scales have been validated against other instruments, showing acceptable convergent and divergent validity [51, 52]. The mean of the scales was used to measure anxiety. Cronbach’s alpha was α = 0.88 for adolescents and α = 0.92 for caregivers.

Depressive symptoms were measured by self-reports of the 9-item Montgomery Åsberg Depression Rating Scale (MADRS-S) [53] and caregiver ratings of the MADRS-P [54]. Items are rated on a scale ranging from 0 to 6, where higher scores indicate more depressive symptoms. Both scales have been validated against other rating instruments showing good concurrent validity [54, 55]. The mean of the scale was used to measure depressive symptoms. Cronbach’s alpha was α = 0.87 for adolescents and α = 0.83 for caregivers.

Conduct problems were assessed by self- and caregiver ratings on items corresponding to the diagnostic criteria of conduct disorder in DSM 5^th^ edition, including all items targeting aggression towards people and animals, destruction of property, deceitfulness or theft and serious violations of rules [1]. The questionnaire comprised 15 items with each item assessing whether a specific event had occurred at any time during the child’s lifespan, rated as 0 (no) or 1 (yes). The sum of the scale was used to measure conduct problems, with a potential range between 0 to 15, where higher scores indicate more life-time conduct problems. Ordinal alpha was 0.95 for adolescents and 0.74 for caregivers.

Autism traits were measured via the Autism-Tics, AD/HD and other Comorbidities inventory, which originated as a telephone interview assessing several psychiatric conditions and symptoms [56]. The interview was utilized as a caregiver-rated questionnaire, where items corresponding to core characteristics of autism were included, specifically the gate items for language, social interaction and flexibility [57]. Items are rated on a scale ranging from 0 (no), 0.5 (yes, to some extent) to 1 (yes), where higher scores indicate the presence of more traits. The mean of the scale was used to measure autism traits. Cronbach’s alpha was α = 0.81.

## Data analyses

Seven adolescents in the diagnostic cohort had missing data on caregiver education level due to caregiver nonresponse and were excluded by pairwise deletion on this variable. One adolescent and one caregiver within the referred population were excluded by pairwise deletion due to complete missing data on ASRS-A and SCAS-S, and MADRS-P respectively. In addition, missing data at the item-level were found for SCAS-S (0.19%) and ASRS-A (0.07%), as well as for SCAS-P (0.35%), ASRS-P (0.21%), MADRS-P (0.63%), the Autism-Tics, AD/HD and other Comorbidities inventory (0.08%) and caregiver-rated conduct problems (0.06%) in the referred cohort. The Little’s MCAR test was significant for ASRS-A and SCAS-S indicating that data were not missing completely at random. However, only one item was missing on ASRS-A and three items were missing on SCAS-S, why imputation was deemed appropriate. Multiple imputation á five repetitions on the item-level for each scale was used and the final data were aggregated in accordance with the Bar Procedure, which combines the separate data files generated by SPSS and averages the imputed values into a single file [58]. The procedure has been used in previous studies [e.g., 59]. Multiple imputation was preferred over other methods as it reduces bias and preserves statistical power [60]. Three outliers, defined as having extreme values of > 3 SD, were identified. Further inspection of the outliers indicated no measurement or data processing errors, why they were kept in the final analyses.

Data were analysed using SPSS version 28. Group differences on demographics were assessed using chi-square tests and Mann-Whitney U tests. Linear regressions were used for the main analyses. Assumptions for regression analyses were met, such as normality of residuals (assessed by visual inspection of histograms and P-P plots of residuals) and no evidence of multicollinearity. Multicollinearity was assessed by examining correlations between independent variables (all correlations were below ≤0.7) and through investigating variance inflation factor (VIF) values (all were below ≤10). Outcome variables were continuous and consisted of reactivity and regulation of sadness, fear, anger and exuberance, in which each emotional construct comprised of two items moderately to largely correlated with each other (see Supplementary Table 1). All independent variables were continuous, except for diagnostic ADHD and sex, which were dichotomous.

We investigated the relation between diagnostic and trait-level ADHD (assessed through ASRS) and self-rated emotion-specific reactivity and regulation, exploring sex-related effects and adjusting for age. We additionally adjusted for other self-rated psychiatric symptoms (symptoms of depression, anxiety and conduct problems) within the referred cohort in a second model. Interaction effects between sex and ADHD were explored on the different facets of reactivity and regulation in separate models using generalized linear models (with normal distribution) while adjusting for age. Caregiver ratings were used in separate models to validate findings, also adjusting for caregiver-rated psychiatric symptoms, including autistic traits. As post-hoc tests, we additionally made adjustments for the diagnostic cohort regarding 1) caregiver-reported diagnoses including co-occurring anxiety conditions and depression, and 2) ADHD-medication. No participants had conduct disorder, why we could not adjust for this. Effect sizes were reported as partial eta-squared (η^2^ₚ) to indicate the proportion of variance in the dependent variable uniquely explained by each predictor and was calculated using general linear models. For partial eta-squared, a η^2^ₚ of 0.01 is regarded as a small effect, a η^2^ₚ of 0.06 as a medium effect, and η^2^ₚ of 0.14 as a large effect. For the linear regressions, adjustments for multiple comparisons were carried out with a Benjamini-Hochberg correction at a false discovery rate of 5% and two tailed tests with *p* < 0.05 were considered statistically significant. Power calculation using g*power 3.1 indicated that, assuming α = 0.05 and power = 0.80, the linear regression models had sufficient sensitivity to detect small-to-moderate effect sizes (f^2^ = 0.08–0.10).

## Results

### Group differences on demographics

Descriptive data on the study variables are presented in Table 2. Individuals with a diagnosis of ADHD had lower caregiver educational level than the comparison group for child- and adolescent (*χ*^*2*^ = 7.88, *p* = 0.005) and caregiver reports (*χ*^*2*^ = 9.08, *p* = 0.003), why educational level was included as a covariate in the analyses for the diagnostic cohort. No differences were found regarding sex (*χ*^*2*^ = 2.82–3.29, *p*s > 0.070), occupational status (*χ*^*2*^ = 0.15–1.78, *p*s > 0.182) or age (*U* = 1220.5–1337.5, *p*s > 0.502) between the groups within the diagnostic cohort.

**Table 2:** Descriptive statistics of self-rated and caregiver-rated emotional reactivity and regulation, as well as co-occurring psychiatric symptoms, across cohorts and informants

	Diagnostic cohort				Referred cohort	
	Self-reports, N = 104		Caregiver reports, N = 103		Self-reports N = 85	Caregiver reports N = 212
	ADHD n = 56	Comp. group n = 48	ADHD n = 55	Comp. group n = 48		
*Reactivity*						
Sadness, m (SD)	3.45 (0.90)	2.44 (0.84)	3.51 (0.85)	2.31 (0.90)	3.52 (1.03)	3.38 (1.03)
Fear, m (SD)	2.90 (1.06)	2.22 (0.73)	3.12 (1.08)	2.13 (0.90)	2.90 (1.22)	2.72 (1.18)
Anger, m (SD)	3.87 (0.95)	2.86 (0.85)	3.45 (1.13)	2.42 (1.04)	3.56 (0.99)	3.47 (1.23)
Exuberance, m (SD)	3.77 (0.75)	3.51 (0.89)	3.52 (0.90)	3.16 (0.88)	3.31 (0.95)	3.16 (1.10)
*Regulation*						
Sadness, m (SD)	2.96 (0.88)	3.33 (0.88)	2.76 (0.60)	3.54 (0.75)	2.72 (0.91)	2.63 (0.90)
Fear, m (SD)	2.90 (0.89)	3.48 (0.81)	2.83 (0.71)	3.69 (0.76)	2.71 (0.99)	2.81 (0.97)
Anger, m (SD)	2.52 (0.97)	3.19 (0.81)	2.79 (0.82)	3.76 (0.81)	2.42 (0.90)	2.71 (0.97)
Exuberance, m (SD)	2.59 (0.81)	3.44 (0.95)	2.88 (0.80)	3.70 (0.92)	2.91 (0.93)	2.93 (1.01)
*Other psychiatric symptoms*						
ADHD symptoms, m (SD)	2.74 (0.68)^a^	1.54 (0.53)^a^	2.65 (0.61)	0.99 (0.56)	2.43 (0.78)	2.14 (0.92)
Depressive symptoms, m (SD)					2.34 (1.09)	1.74 (0.96)
Anxiety, m (SD)					1.10 (0.54)	0.72 (0.42)
Conduct problems, m (SD)					2.15 (3.07)	1.47 (1.88)
Autism traits, m (SD)						0.24 (0.17)

*Note*: ADHD: attention-deficit/hyperactivity disorder; Comp. group: comparison group. A higher score of reactivity indicates more intensity/frequency of the emotion, whereas a higher score of regulation indicates a better ability to regulate one’s emotions

^a^ Only completed by a subsample of adolescents 13–18 years (*n* = 62)

### ADHD and emotional reactivity

As shown in Table 3, diagnostic ADHD was associated with higher levels of sadness, fear and anger, whereas trait-level ADHD was positively related to reactivity of anger and exuberance, adjusting for age, sex and other psychiatric symptoms. Large effect sizes (η^2^ₚ) were observed for the association between an ADHD diagnosis and reactivity to sadness and anger. For trait-level ADHD, moderate to large effect sizes were found for the relation with anger and exuberance reactivity. In the diagnostic cohort, females scored higher on reactivity on all negative emotions, whereas no significant sex-related effects were found for the referred cohort in the fully adjusted model. No interaction effects of sex by diagnostic or trait-level ADHD were identified. Caregiver ratings largely validated the primary findings, see Supplementary Table S2.

**Table 3:** Associations between diagnostic- and trait-level ADHD and self-rated emotion-specific reactivity

	Sadness reactivity				Fear reactivity				Anger reactivity				Exuberance reactivity			
	B (SE B)	β	p	η^2^ₚ	B (SE B)	β	p	η^2^ₚ	B (SE B)	β	p	η^2^ₚ	B (SE B)	β	p	η^2^ₚ
*Diagnostic cohort* (*N* = 104)																
Model 1																
ADHD (diagnostic)	1.00 (0.17)	0.50	** < 0.001**	0.29	0.68 (0.19)	0.35	** < 0.001**	0.13	0.90 (0.19)	0.44	** < 0.001**	0.21	0.19 (0.18)	0.12	0.272	0.02
Sex (female)	0.69 (0.16)	0.34	** < 0.001**	0.13	0.55 (0.19)	0.28	**0.004**	0.06	0.63 (0.18)	0.31	** < 0.001**	0.09	0.27 (0.17)	0.17	0.120	0.03
Age	0.07 (0.04)	0.15	0.069	0.06	0.01 (0.04)	0.02	0.870	0.01	−0.03 (0.04)	−0.06	0.498	0.00	0.01 (0.04)	0.03	0.779	0.01
Educational level (*n* = 97)^a^	0.39 (0.20)	0.17	0.047	0.04	0.38 (0.23)	0.16	0.097	0.03	−0.03 (0.22)	−0.01	0.896	0.00	−0.08 (0.21)	−0.04	0.699	0.00
Model 1, R^2^_adj_	0.41				0.19				0.30				0.02			
*Referred cohort* (*N* = 85)																
Model 1																
ADHD (trait)	0.08 (0.14)	0.06	0.593	0.00	0.29 (0.16)	0.19	0.081	0.04	0.54 (0.13)	0.43	** < 0.001**	0.18	0.42 (0.13)	0.35	**0.002**	0.12
Sex (female)	0.87 (0.27)	0.35	**0.002**	0.11	0.94 (0.31)	0.32	**0.003**	0.10	0.19 (0.24)	0.08	0.440	0.01	0.22 (0.25)	0.10	0.366	0.01
Age	0.01 (0.06)	0.02	0.868	0.00	−0.05 (0.07)	−0.07	0.471	0.01	−0.10 (0.06)	−0.18	0.069	0.03	0.02 (0.06)	0.04	0.734	0.00
Model 1, R^2^_adj_	0.10				0.14				0.22				0.11			
Model 2																
ADHD (trait)	−0.09 (0.11)	−0.07	0.409	0.01	0.02 (0.13)	0.01	0.909	0.00	0.41 (0.13)	0.32	**0.003**	0.11	0.45 (0.14)	0.37	**0.002**	0.12
Sex (female)	0.43 (0.20)	0.17	0.036	0.05	0.41 (0.24)	0.14	0.089	0.04	0.03 (0.24)	0.01	0.896	0.00	0.26 (0.25)	0.11	0.301	0.01
Age	−0.02 (0.05)	−0.04	0.598	0.01	−0.07 (0.05)	−0.10	0.184	0.03	−0.11 (0.06)	−0.20	0.040	0.05	0.03 (0.06)	0.06	0.594	0.00
Depressive symptoms (*n* = 84)^b^	0.47 (0.09)	0.50	** < 0.001**	0.25	−0.07 (0.11)	−0.06	0.559	0.01	0.09 (0.11)	0.09	0.452	0.02	−0.26 (0.12)	−0.30	0.027	0.07
Anxiety (*n* = 84)^b^	0.53 (0.20)	0.28	**0.008**	0.09	1.61 (0.24)	0.72	** < 0.001**	0.38	0.39 (0.24)	0.21	0.107	0.02	0.26 (0.25)	0.15	0.303	0.02
Conduct problems	−0.01 (0.03)	−0.02	0.835	0.00	−0.02 (0.03)	−0.05	0.549	0.00	0.04 (0.03)	0.12	0.245	0.02	−0.03 (0.03)	−0.09	0.421	0.01
Model 2, R^2^_adj_	0.54				0.52				0.27				0.15			

*Bold*: Significant result after Benjamini-Hochberg correction. Note: ADHD: attention-deficit/hyperactivity disorder; sex = assigned sex at birth. A higher score of reactivity indicates more intensity/frequency of the emotion. Pairwise deletion was employed for the models, except for when calculating η^2^ ₚ, which used listwise deletion

^a^ Due to missing data on caregiver-rated educational level, the sample size for this variable was reduced to 97 participants

^b^ Complete missingness on ADHD and anxiety symptoms for one participant limited the referred cohort to 84 participants for these variables

### Emotion regulation

As shown in Table 4, diagnostic ADHD was negatively associated with regulation of fear, anger and exuberance. Trait-level ADHD was related to lower regulation of anger and exuberance, when adjusting for age, sex and other psychiatric symptoms. Overall, η^2^ ₚ values were generally smaller for regulation measures compared to reactivity measures. Moderate to large effects were observed for both diagnostic and trait-level ADHD in relation to regulation of anger and exuberance. No age- or sex-related patterns were found. Findings were validated in the caregiver ratings, see Supplementary Table S3. Notable differences from the self-reports were that diagnostic ADHD was additionally associated with sadness dysregulation (β = −50, *p* < 0.001).

**Table 4:** Associations between diagnostic-and trait-level ADHD and self-rated emotion-specific regulation

	Sadness regulation				Fear regulation				Anger regulation				Exuberance regulation			
	B (SE B)	β	p	η^2^ₚ	B (SE B)	β	p	η^2^ₚ	B (SE B)	β	p	η^2^ₚ	B (SE B)	β	p	η^2^ₚ
*Diagnostic cohort* (*N* = 104)																
Model 1																
ADHD (diagnostic)	−0.39 (0.19)	−0.22	0.042	0.05	−0.53 (0.18)	−0.30	**0.005**	0.11	−0.69 (0.19)	−0.36	** < 0.001**	0.13	−0.86 (0.19)	−0.45	** < 0.001**	0.19
Sex (female)	−0.28 (0.19)	−0.16	0.134	0.01	−0.29 (0.18)	−0.16	0.116	0.01	−0.22 (0.19)	−0.11	0.261	0.01	−0.21 (0.19)	−0.11	0.267	0.01
Age	−0.05 (0.04)	−0.13	0.191	0.03	0.01 (0.04)	0.02	0.851	0.00	0.01 (0.04)	0.02	0.809	0.00	0.07 (0.04)	0.16	0.092	0.03
Educational level (*n* = 97)^a^	−0.21 (0.22)	−0.10	0.353	0.01	−0.02 (0.22)	−0.01	0.926	0.00	−0.23 (0.23)	−0.10	0.314	0.01	−0.23 (0.22)	−0.10	0.312	0.01
Model 1, R^2^_adj_	0.06				0.09				0.11				0.19			
*Referred cohort* (*N* = 85)																
Model 1																
ADHD (trait)	−0.08 (0.13)	−0.07	0.567	0.00	−0.21 (0.14)	−0.16	0.157	0.03	−0.47 (0.12)	−0.41	** < 0.001**	0.16	−0.39 (0.13)	−0.32	**0.004**	0.10
Sex (female)	−0.31 (0.25)	−0.14	0.213	0.02	−0.04 (0.27)	−0.02	0.896	0.00	−0.12 (0.23)	−0.06	0.592	0.00	0.32 (0.25)	0.14	0.202	0.02
Age	0.05 (0.06)	0.10	0.358	0.01	−0.01 (0.06)	−0.02	0.874	0.00	−0.05 (0.05)	−0.10	0.319	0.01	−0.04 (0.06)	−0.07	0.534	0.00
Model 1, R^2^_adj_	0.00				−0.01				0.15				0.07			
Model 2																
ADHD (trait)	0.02 (0.13)	0.02	0.860	0.00	−0.13 (0.16)	−0.11	0.389	0.01	−0.40 (0.12)	−0.35	**0.002**	0.12	−0.52 (0.14)	−0.43	** < 0.001**	0.15
Sex (female)	−0.12 (0.23)	−0.05	0.605	0.01	0.12 (0.28)	0.05	0.665	0.00	−0.01 (0.22)	−0.00	0.979	0.00	0.23 (0.25)	0.10	0.371	0.01
Age	0.08 (0.05)	0.14	0.141	0.02	0.00 (0.06)	0.01	0.947	0.00	−0.04 (0.05)	−0.07	0.457	0.01	−0.04 (0.06)	−0.07	0.514	0.00
Depressive symptoms (*n* = 84)^b^	−0.43 (0.11)	−0.51	** < 0.001**	0.19	−0.23 (0.13)	−0.25	0.078	0.04	−0.33 (0.10)	−0.39	**0.002**	0.11	−0.10 (0.12)	−0.12	0.400	0.01
Anxiety (*n* = 84)^b^	0.06 (0.23)	0.03	0.805	0.00	−0.11 (0.27)	−0.06	0.688	0.00	0.12 (0.22)	0.07	0.596	0.00	0.50 (0.25)	0.29	0.050	0.05
Conduct problems	−0.03 (0.03)	−0.11	0.270	0.01	−0.01 (0.04)	−0.04	0.757	0.00	−0.03 (0.03)	−0.11	0.259	0.02	0.04 (0.03)	0.13	0.240	0.02
Model 2, R^2^_adj_	0.23				0.04				0.27				0.10			

*Bold*: Significant result after Benjamini-Hochberg correction. Note: ADHD: attention-deficit/hyperactivity disorder; sex = assigned sex at birth. A higher score of reactivity indicates more intensity/frequency of the emotion. Pairwise deletion was employed for the models, except for when calculating η^2^ ₚ, which used listwise deletion

^a^ Due to missing data on caregiver-rated educational level, the sample size for this variable was reduced to 97 participants

^b^ Complete missingness on ADHD and anxiety symptoms for one participant limited the referred cohort to 84 participants for these variables

### Post-hoc analyses

Post-hoc analyses were conducted to investigate the robustness of the results in the diagnostic cohort. These included adjustments for caregiver-reported co-occurring diagnostic conditions of anxiety and depression, as well as for ADHD medication, in separate models. Findings remained largely the same, albeit one notable difference was that the association between ADHD and fear regulation became non-significant in self-ratings after adjusting for medication (β = −18, *p* = 0.112). See Supplementary Tables S4–S7 for details.

## Discussion

In this cross-sectional study, we investigated associations between both diagnostic and trait-level ADHD and self-rated reactivity and regulation of different emotions, which to the best of our knowledge has not been done before. We found that a diagnosis of ADHD was associated with elevated reactivity of all negative emotions when including age and sex in the model, whereas trait-level ADHD was related to higher levels of reactivity of anger and exuberance when additionally adjusting for other psychiatric symptoms. Further, diagnostic ADHD was linked to lower regulation of fear, anger and exuberance, whereas trait-level ADHD was associated with lower regulation of anger and exuberance in the fully adjusted model. Findings were validated in caregiver ratings and remained largely consistent in post-hoc analyses where we accounted for co-occurring anxiety and depression, as well as ADHD medication, in the diagnostic cohort.

First and foremost, diagnostic and trait-level ADHD were robustly linked to higher levels of reactivity and dysregulation of anger across informants. The findings are in line with our hypotheses and with recent research showing that irritability and anger may play an important role in ADHD [30, 33]. Indeed, recent profiling of subgroups in children aged 7–12 years with a diagnosis of ADHD indicated that anger in particular may predict a worse clinical prognosis [30]. Further aligned with our hypothesis were the significant associations of both diagnostic ADHD and trait-level ADHD with exuberance, although these observations were not as robust. The associations varied across groups, where diagnostic ADHD was related to dysregulation of exuberance, but not reactivity, a result inconsistent with our initial hypothesis. Conversely, others have found a stronger link between elevated positive emotional reactivity and diagnostic ADHD compared to other clinical groups [34]. As Okado and colleagues [34] considered a wider range of positive emotions, it is possible that our narrower focus on exuberance may not be as indicative for ADHD. Alternatively, dysregulation of exuberance may be a more prominent characteristic than having elevated exuberance reactivity for the ADHD population, at least when compared to typically developing controls. Moreover, our measure of exuberance reactivity had lower inter-item correlations within the diagnostic cohort compared to the referred cohort, suggesting potential methodological problems. Thus, further investigating positive emotions in ADHD is necessary, while also increasing precision by specifying the conceptualizations of exuberance and other similar constructs.

The relation between ADHD and sadness, and fear, paints a more ambiguous picture. Diagnostic ADHD was related to elevated reactivity of sadness and fear, as well as fear dysregulation, whereas trait-level ADHD was not related to any measures of sadness or fear. Conversely, previous research suggests that trait-level ADHD is associated with reactivity (but not dysregulation) of fear and sadness in non-clinical children [7]. However, the authors did not adjust for other psychiatric symptoms, which may act as potential confounders. Interestingly, in the referred cohort, depressive symptoms were associated with higher reactivity and lower dysregulation of sadness, whereas anxiety was only positively related to sadness reactivity. Further, anxiety was uniquely related to increased fear reactivity. Additionally, caregiver reports suggest that autism traits are associated with increased sadness reactivity and conduct problems with lower sadness regulation. Possibly, co-occurring psychiatric traits and symptoms may partly account for the relation between diagnostic ADHD and reactivity and regulation of sadness and fear. While post-hoc analyses indicate that results largely remained consistent after accounting for co-occurring anxiety and depression, these findings must be interpreted with caution, given that the co-occurring conditions were based on caregiver reports. Nonetheless, our findings highlight the need for greater specificity in the field and to include co-occurring psychiatric symptoms when investigating emotional difficulties in ADHD. It may also be vital to refine comorbidity measures. Combining anxiety and depressive symptoms into a broader internalizing construct, as often done, may not be advisable, as our results suggest this could mask distinct associations.

Females displayed higher intensity and frequency, but not regulation, of sadness, fear and anger in the diagnostic cohort. This finding may reflect gender differences in emotional awareness and expression, as adolescent females have been reported to express emotions to a greater extent than males [61], possibly aligning with socialization theories suggesting that girls are expected to be more emotionally expressive than boys [62]. In the Swedish context, where gender equality and emotional openness may be culturally emphasized, such differences might be somewhat attenuated, yet traditional gender norms could still influence how boys and girls are encouraged to express emotions. Such sex differences were not reflected in the fully adjusted model of the referred cohort. Possibly, these mixed findings could partly be explained by co-occurring psychiatric traits. Alternatively, since the referred cohort had a very high percentage of females (78.8%), sex-related effects could be more difficult to discern. The association between diagnostic and trait-level ADHD with emotional reactivity and regulation was similar for both females and males, which reflect previous research [23, 45]. To further disentangle sex-related effects and developmental patterns, it seems important to simultaneously examine these factors alongside co-occurring psychiatric traits. Age was not related to emotional reactivity or regulation which is a surprising finding given that childhood and adolescence are periods with an increase in negative emotions, alongside a transition to more internal regulatory skills. At the same time, previous findings om emotion regulation during these periods have been mixed, where both reactivity and regulation may be affected by different emotions and where the association with age may not be linear [26]. It should also be noted that the present study included a relatively small sample with a limited age range, which may have reduced the ability to detect more subtle or non-linear age effects. Future research should include larger samples during specific developmental periods in order to investigate how reactivity and regulation of emotions may differ for various age groups.

Findings need to be regarded in the context of strengths and limitations of the current study. Limitations include reliance on self-reports for the outcome measures, which may introduce bias, such as limited self-awareness or a tendency to report socially desirable responses. Although adolescent mental health problems, specifically emotional problems and internalizing symptoms, are often measured with self-ratings [63], we tried to account for this limitation by validating findings through caregiver ratings. However, caregiver reports may also introduce informant bias, as caregivers’ perceptions can be shaped by their own experiences or interpretations of the child’s behaviour. Thus, although caregiver data offset some self-report limitations, potential bias cannot be ruled out. Additionally, in the referred cohort, both the predictor and outcome variables were measured using the same method (i.e., adolescent self-reports), which may result in a common method bias inflating the associations. In contrast, in the diagnostic cohort, the outcome variable was diagnostic status, limiting the risk of such bias. Each emotion-specific reactivity and regulation measure only encompassed two items, which may have compromised the reliability and validity of our assessments. However, the EQ is a well-known instrument that has been previously utilized in ADHD research [7, 22, 23]. Further, we did not conduct a broader diagnostic assessment of the comparison group in the diagnostic cohort, albeit we administered the ADHD module of MINI-Kid for a more thorough screening of ADHD. Several analyses were carried out in order to investigate emotion-specific reactivity and regulation which increases the risk of type I errors. Post-hoc corrections for multiple comparisons were made for the analyses to counteract this risk. Moreover, due to limited statistical power, we did not adjust for co-occurring psychiatric symptoms in the main analyses for the diagnostic cohort. However, we included post-hoc analyses, where we adjusted for caregiver-reported co-occurring anxiety and depression, and the results were largely consistent with the original findings. As the caregiver-reported diagnoses were not validated through medical records or diagnostic assessments, and since a limited number of participants had reported depression (*n* = 3) or an anxiety condition (*n* = 10) resulting in lower statistical power, findings must be interpreted with caution and more research using larger samples is necessary. Further, with a larger sample size it would also be possible to more thoroughly explore three-way interaction effects such as between diagnosis, sex and age. We did not adjust for oppositional defiant disorder (ODD), common in ADHD [64] and partly characterized by emotion dysregulation [1], which may have influenced our results. However, including ODD may also risk adjusting for the very constructs we aim to study given its strong overlap with negative emotionality, particularly anger. Therefore, we included conduct problems, which encompass defiant, aggressive and disobedient behaviours, without involving emotional traits. More than a third of the participants in the diagnostic cohort had ADHD medication, which could potentially weaken the strengths of the observed associations, as ADHD medication might alleviate emotional symptoms [3]. At the same time, post-hoc analyses suggest that findings largely remained the same when accounting for ADHD medication. A majority of the participants with an ADHD diagnosis were females (63.8%), which does not reflect the sex distribution of ADHD [1]. The referred cohort displayed an even more unbalanced sex ratio (78.8% females). As Meyer and colleagues [65] points out, this may reflect a self-selection bias in which girls are more likely to partake in research, which may differentiate them from boys who decline participation. Lastly, the generalizability of the findings may be hampered since both samples were recruited from a university town in Sweden, as well as due to the specific exclusion criteria of the diagnostic cohort, such as intellectual disability. Strengths of the study comprise the inclusion of two distinct samples, including a thoroughly assessed diagnostic cohort. Albeit the samples may differ in unassessed aspects, the inclusion of two samples allows for the replication and validation of findings. Further, we investigated how both diagnostic and trait-level ADHD relate to reactivity and regulation of different emotions in child- and adolescent populations, which expands previous research by increasing specificity and validating findings using ratings from both the children/adolescents and their caregivers. We also considered common co-occurring psychiatric symptoms in ADHD known to be associated with emotional reactivity and dysregulation.

## Conclusions

The current study extends previous research by specifying the associations between ADHD and emotion-specific reactivity and regulation. By addressing important potential confounders, it offers a more comprehensive insight into the emotional difficulties linked to ADHD. Moreover, examining both diagnostic ADHD in a clinical phenotyped sample and trait-level ADHD in a sample referred to a CAP unit for psychiatric difficulties may provide a more nuanced understanding of the condition. Findings suggest that ADHD is robustly associated with reactivity and dysregulation of anger. Thus, it appears clinically important to address anger during assessment as a possible indicator of ADHD-related impairment, since anger dysregulation has been linked to oppositional behaviour, interpersonal conflict, substance abuse and negatively impacts mental health [66]. For treatment, interventions targeting emotion regulation skills may be prioritized when anger dysregulation is prominent. While both reactivity and dysregulation of exuberance was associated with trait-level ADHD, dysregulation of exuberance may be more characteristic for diagnostic ADHD. Future studies should utilize different measures in order to elucidate which aspects of positive emotions are most relevant to ADHD. Further, accounting for co-occurring psychiatric traits seems crucial when investigating emotional reactivity and dysregulation in ADHD. In particular, anxiety and depressive symptoms may be more important for reactivity of sadness and fear, as well as dysregulation of sadness, than trait-level ADHD. More research is needed to clarify whether these effects may extend to diagnostic ADHD. Females had higher levels of negative emotional reactivity in the diagnostic cohort, but no interaction effects between sex and ADHD were identified, suggesting that the association between ADHD and emotional reactivity does not differ between females and males.

## Electronic supplementary material

Below is the link to the electronic supplementary material.

Supplementary Material 1

## Data Availability

Consent for sharing individual data outside the research team was not obtained. However, reasonable requests for patient-level data can be made to the corresponding author (RA) and will be considered after discussion with the ethical review board. Relevant data are included in the manuscript or supplementary information files.

## Abbreviations

ADHD: Attention-deficit/hyperactivity disorder
ASRS: ADHD Self-Report Scale for adolescents (ASRS)
CAP outpatient unit: Child and adolescent psychiatric outpatient unit
EPIQ: Electronic Psychiatric Intake Questionnaire
EQ: Emotion Questionnaire
MADRS: Montgomery Åsberg Depression Rating Scale
MCAR: Missing completely at random
MINI-Kid: Mini-International Neuropsychiatric Interview for Children and Adolescents
ODD: Oppositional defiant disorder
SCAS: Spence Children’s Anxiety Scale
