# Supplementary material for: Reactivity and regulation of negative and positive emotions in child- and adolescent diagnostic and trait-level ADHD: a cross-sectional study
Source: BMC Psychiatry. 2025 Dec 23;26:68. doi: 10.1186/s12888-025-07708-0 (PMC12837954; doi:10.1186/s12888-025-07708-0)
Supplement: Supplementary file 1 — Supplementary Material 1 [file 12888_2025_7708_MOESM1_ESM.docx]

**Supplementary information**

**Table S1.** Emotion-specific item intercorrelations for reactivity (item a and b) and regulation (item c and d) for self-reports and caregiver reports within the diagnostic cohort and the referred cohort

|  | **Diagnostic cohort** | | **Referred cohort** | |
| --- | --- | --- | --- | --- |
|  | Self-reports | Caregiver ratings | Self-reports | Caregiver ratings |
| 1. Sadness reactivity (a – b) | .53** | .67** | .53** | .36** |
| 2. Sadness regulation (c – d) | .44** | .62** | .52** | .52** |
| 3. Fear reactivity (a – b) | .42** | .71** | .70** | .60** |
| 4. Fear regulation (c – d) | .37** | .61** | .56** | .57** |
| 5. Anger reactivity (a – b) | .51** | .74** | .53** | .70** |
| 6. Anger regulation (c – d) | .43** | .65** | .57** | .55** |
| 7. Exuberance reactivity (a – b) | .37** | .58** | .51** | .63** |
| 8. Exuberance regulation (c – d) | .46** | .66** | .53** | .63** |
| ** = Significant at the .01-level (Spearman’s, two-tailed) | | | | |

**Table S2.** Associations between diagnostic-and trait-level ADHD and emotion-specific reactivity for caregiver reports

|  | Sadness reactivity | | | | Fear reactivity | | | | Anger reactivity | | | | Exuberance reactivity | | | |
| --- | --- | --- | --- | --- | --- | --- | --- | --- | --- | --- | --- | --- | --- | --- | --- | --- |
|  | B (SE B) | β | p | R^2^ _adj_ | B (SE B) | β | p | R^2^ _adj_ | B (SE B) | β | p | R^2^ _adj_ | B (SE B) | β | p | R^2^ _adj_ |
| ***Diagnostic cohort*** (N=103) |  |  |  |  |  |  |  |  |  |  |  |  |  |  |  |  |
| Model 1 |  |  |  | .34 |  |  |  | .22 |  |  |  | .23 |  |  |  | .01 |
| ADHD (diagnostic) | 1.15 (.18) | .54 | **<.001** |  | .96 (.21) | .43 | **<.001** |  | .86 (.22) | .36 | **<.001** |  | .34 (.19) | .19 | .078 |  |
| Sex (female) | .33 (.18) | .16 | .064 |  | .44 (.20) | .20 | .032 |  | .43 (.22) | .18 | .052 |  | -.09 (.18) | -.05 | .637 |  |
| Age | -.08 (.04) | -.18 | .029 |  | -.05 (.04) | -.11 | .240 |  | -.12 (.05) | -.23 | **.013** |  | -.03 (.04) | -.07 | .518 |  |
| Education level | .16 (.22) | .06 | .464 |  | .26 (.25) | .10 | .293 |  | -.19 (.27) | -.07 | .476 |  | -.13 (.23) | -.06 | .577 |  |
|  |  |  |  |  |  |  |  |  |  |  |  |  |  |  |  |  |
| ***Referred cohort*** (N=212) |  |  |  |  |  |  |  |  |  |  |  |  |  |  |  |  |
| Model 1 |  |  |  | .02 |  |  |  | -.01 |  |  |  | .21 |  |  |  | .17 |
| ADHD (trait) | .18 (.08) | .16 | .031 |  | -.04 (.10) | -.03 | .718 |  | .45 (.09) | .33 | **<.001** |  | .34 (.08) | .28 | **<.001** |  |
| Sex (female) | .19 (.16) | .09 | .246 |  | -.03 (.19) | -.01 | .882 |  | .05 (.17) | .02 | .786 |  | .09 (.16) | .04 | .586 |  |
| Age | -.02 (.03) | -.06 | .449 |  | .02 (.03) | .04 | .623 |  | -.09 (.03) | -.23 | **.002** |  | -.08 (.03) | -.24 | **.001** |  |
|  |  |  |  |  |  |  |  |  |  |  |  |  |  |  |  |  |
| Model 2 |  |  |  | .33 |  |  |  | .43 |  |  |  | .27 |  |  |  | .22 |
| ADHD (trait) | -.00 (.08) | -.00 | .986 |  | -.10 (.08) | -.08 | .217 |  | .30 (.09) | .23 | **<.001** |  | .42 (.09) | .35 | **<.001** |  |
| Sex (female) | -.03 (.14) | -.02 | .814 |  | -.41 (.14) | -.17 | **.005** |  | -.01 (.17) | -.01 | .944 |  | .17 (.16) | .07 | .288 |  |
| Age | -.03 (.02) | -.08 | .274 |  | .01 (.02) | .02 | .720 |  | -.07 (.03) | -.19 | **.012** |  | -.08 (.03) | -.23 | **.004** |  |
| Depressive symptoms | .44 (.08) | .41 | **<.001** |  | .08 (.08) | .06 | .323 |  | .22 (.09) | .17 | **.020** |  | -.24 (.09) | -.21 | **.005** |  |
| Anxiety | .32 (.18) | .13 | .072 |  | 1.80 (.19) | .64 | **<.001** |  | -.11 (.22) | -.04 | .612 |  | .02 (.21) | .01 | .935 |  |
| Conduct problems | .06 (.04) | .10 | .118 |  | .01 (.04) | .02 | .728 |  | .14 (.05) | .21 | **.003** |  | -.08 (.04) | -.14 | .051 |  |
| Autism traits | 1.33 (.42) | .21 | **.002** |  | .28 (.44) | .04 | .529 |  | .67 (.53) | .09 | .206 |  | .26 (.49) | .04 | .596 |  |
| *Bold*: Significant result after Benjamini-Hochberg correction. *Note*: ADHD: attention-deficit/hyperactivity disorder; sex = assigned sex at birth. A higher score of reactivity indicates more intensity/frequency of the emotion, whereas a higher score of regulation indicates a better ability to regulate one’s emotions. Complete missingness on depressive symptoms for one caregiver in the referred cohort limited the sample size to 211 participants for these variables. | | | | | | | | | | | | | | | | |

**Table S3.** Associations between diagnostic-and trait-level ADHD and emotion-specific regulation for caregiver reports

|  | Sadness regulation | | | | Fear regulation | | | | Anger regulation | | | | Exuberance regulation | | | |
| --- | --- | --- | --- | --- | --- | --- | --- | --- | --- | --- | --- | --- | --- | --- | --- | --- |
|  | B (SE B) | β | p | R^2^ _adj_ | B (SE B) | β | p | R^2^ _adj_ | B (SE B) | β | p | R^2^ _adj_ | B (SE B) | β | p | R^2^ _adj_ |
| ***Diagnostic cohort*** (N=103) |  |  |  |  |  |  |  |  |  |  |  |  |  |  |  |  |
| Model 1 |  |  |  | .23 |  |  |  | .24 |  |  |  | .24 |  |  |  | .16 |
| ADHD (diagnostic) | -.78 (.14) | -.50 | **<.001** |  | -.85 (.16) | -.50 | **<.001** |  | -.91 (.17) | -.48 | **<.001** |  | -.78 (.18) | -.41 | **<.001** |  |
| Sex (female) | -.07 (.14) | -.05 | .619 |  | -.12 (.15) | -.07 | .444 |  | -.09 (.17) | -.05 | .606 |  | -.12 (.18) | -.06 | .499 |  |
| Age | .00 (.03) | .01 | .938 |  | .04 (.03) | .10 | .280 |  | .02 (.04) | .04 | .622 |  | .04 (.04) | .09 | .336 |  |
| Education level | -.04 (.17) | -.02 | .812 |  | -.10 (.19) | -.05 | .589 |  | .17 (.21) | .07 | .423 |  | .01 (.22) | -.02 | .982 |  |
|  |  |  |  |  |  |  |  |  |  |  |  |  |  |  |  |  |
| ***Referred cohort*** (N=212) |  |  |  |  |  |  |  |  |  |  |  |  |  |  |  |  |
| Model 1 |  |  |  | .05 |  |  |  | .01 |  |  |  | .10 |  |  |  | .22 |
| ADHD (trait) | -.04 (.07) | -.05 | .539 |  | -.10 (.08) | -.09 | .226 |  | -.33 (.08) | -.32 | **<.001** |  | -.45 (.07) | -.41 | **<.001** |  |
| Sex (female) | -.51 (.14) | -.27 | **<.001** |  | -.23 (.15) | -.12 | .123 |  | -.25 (.14) | -.13 | .083 |  | -.22 (.14) | -.11 | .117 |  |
| Age | .01 (.02) | .03 | .728 |  | .02 (.02) | .06 | .470 |  | .02 (.02) | .07 | .350 |  | .05 (.02) | .17 | **.018** |  |
|  |  |  |  |  |  |  |  |  |  |  |  |  |  |  |  |  |
| Model 2 |  |  |  | .21 |  |  |  | .12 |  |  |  | .20 |  |  |  | .25 |
| ADHD (trait) | .06 (.07) | .06 | .406 |  | .01 (.08) | .01 | .911 |  | -.19 (.08) | -.19 | **.012** |  | -.37 (.08) | -.34 | **<.001** |  |
| Sex (female) | -.35 (.13) | -.19 | **.007** |  | -.09 (.15) | -.04 | .554 |  | -.19 (.14) | -.09 | .184 |  | -.20 (.14) | -.10 | .154 |  |
| Age | .01 (.02) | .04 | .572 |  | .02 (.03) | .06 | .438 |  | .01 (.02) | .04 | .646 |  | .05 (.02) | .14 | .060 |  |
| Depressive symptoms | -.25 (.07) | -.27 | **<.001** |  | -.23 (.08) | -.23 | **.005** |  | -.23 (.08) | -.23 | **.002** |  | -.12 (.08) | -.11 | .136 |  |
| Anxiety | -.37 (.17) | -.17 | .029 |  | -.35 (.19) | -.15 | .073 |  | .12 (.18) | .05 | .499 |  | .11 (.19) | .05 | .562 |  |
| Conduct problems | -.09 (.03) | -.19 | **.008** |  | -.07 (.04) | -.14 | .059 |  | -.08 (.04) | -.16 | .026 |  | -.11 (.04) | -.21 | **.003** |  |
| Autism traits | .12 (.40) | .02 | .766 |  | -.28 (.45) | -.05 | .543 |  | -1.12 (.43) | -.19 | **.010** |  | .13 (.44) | .02 | .772 |  |
| *Bold*: Significant result after Benjamini-Hochberg correction. *Note*: ADHD: attention-deficit/hyperactivity disorder; sex = assigned sex at birth. A higher score of reactivity indicates more intensity/frequency of the emotion, whereas a higher score of regulation indicates a better ability to regulate one’s emotions. Complete missingness on depressive symptoms for one caregiver in the referred cohort limited the sample size to 211 participants for these variables. | | | | | | | | | | | | | | | | |

**Table S4**. Post-hoc analyses of associations between diagnostic- and trait-level ADHD and self-rated emotion-specific reactivity, while adjusting for co-occurring psychiatric symptoms and medication, respectively

|  | Sadness reactivity | | | | Fear reactivity | | | | Anger reactivity | | | | Exuberance reactivity | | | |
| --- | --- | --- | --- | --- | --- | --- | --- | --- | --- | --- | --- | --- | --- | --- | --- | --- |
|  | B (SE B) | β | p | R^2^ _adj_ | B (SE B) | β | p | R^2^ _adj_ | B (SE B) | β | p | R^2^ _adj_ | B (SE B) | β | p | R^2^ _adj_ |
| ***Diagnostic cohort*** (N=104) |  |  |  |  |  |  |  |  |  |  |  |  |  |  |  |  |
|  |  |  |  |  |  |  |  |  |  |  |  |  |  |  |  |  |
| Comorbidity model |  |  |  | .41 |  |  |  | .18 |  |  |  | .32 |  |  |  | .01 |
| ADHD (diagnostic) | .96 (.17) | .48 | **<.001** |  | .62 (.20) | .31 | **.003** |  | .93 (.19) | .45 | **<.001** |  | .26 (.19) | .16 | .173 |  |
| Sex (female) | .64 (.17) | .32 | **<.001** |  | .50 (.19) | .25 | **.011** |  | .61 (.18) | .29 | **.001** |  | .30 (.18) | .18 | .097 |  |
| Age | .07 (.04) | .16 | .060 |  | .00 (.04) | .01 | .931 |  | -.01 (.04) | -.03 | .754 |  | .02 (.04) | .05 | .649 |  |
| Educational level | .36 (.20) | .15 | .072 |  | .34 (.23) | .15 | .141 |  | -.05 (.22) | -.02 | .822 |  | -.06 (.21) | -.03 | .781 |  |
| Anxiety conditions | .38 (.29) | .11 | .201 |  | .44 (.34) | .14 | .191 |  | .17 (.32) | .05 | .608 |  | -.27 (.31) | -.10 | .393 |  |
| Depression | -.64 (.49) | -.11 | .190 |  | -.27 (.56) | -.05 | .631 |  | -1.15 (.54) | -.19 | .036 |  | -.19 (.52) | -.04 | .720 |  |
|  |  |  |  |  |  |  |  |  |  |  |  |  |  |  |  |  |
| Medication model |  |  |  | .40 |  |  |  | .19 |  |  |  | .30 |  |  |  | .01 |
| ADHD (diagnostic) | .99 (.19) | .50 | **<.001** |  | .56 (.22) | .29 | **.011** |  | .83 (.21) | .40 | **<.001** |  | .25 (.20) | .15 | .224 |  |
| Sex (female) | .70 (.17) | .34 | **<.001** |  | .55 (.19) | .28 | **.005** |  | .63 (.18) | .31 | **<.001** |  | .27 (.18) | .17 | .121 |  |
| Age | .07 (.04) | .15 | .072 |  | .00 (.04) | .01 | .957 |  | -.03 (.04) | -.07 | .463 |  | .01 (.04) | .03 | .744 |  |
| Educational level | .39 (.20) | .17 | .048 |  | .38 (.22) | .17 | .090 |  | -.03 (.22) | -.01 | .911 |  | -.08 (.21) | -.04 | .689 |  |
| Medication | -.01 (.22) | -.00 | .975 |  | .29 (.24) | .13 | .234 |  | .16 (.24) | .07 | .502 |  | -.12 (.23) | -.06 | .593 |  |
| *Bold*: Significant result after Benjamini-Hochberg correction. *Note:* ADHD: attention-deficit/hyperactivity disorder; sex = assigned sex at birth. A higher score of reactivity indicates more intensity/frequency of the emotion, whereas a higher score of regulation indicates a better ability to regulate one’s emotions. Anxiety conditions include caregiver-reported diagnoses of unspecified anxiety, social anxiety disorder, generalized anxiety disorder and post-traumatic stress disorder. Ten participants with ADHD had co-occurring anxiety conditions and three participants with ADHD had co-occurring depression. A total of 23 participants with ADHD had ADHD medication (91.3% stimulants). Note that two participants with ADHD and five typically developing individuals had missing data on caregiver-reported diagnoses and medication, resulting in a reduced sample size for these variables (n=97) using pairwise deletion. | | | | | | | | | | | | | | | | |

**Table S5**. Post-hoc analyses of associations between diagnostic- and trait-level ADHD and self-rated emotion-specific regulation, while adjusting for co-occurring psychiatric symptoms and medication, respectively

|  | Sadness regulation | | | | Fear regulation | | | | Anger regulation | | | | Exuberance regulation | | | |
| --- | --- | --- | --- | --- | --- | --- | --- | --- | --- | --- | --- | --- | --- | --- | --- | --- |
|  | B (SE B) | β | p | R^2^ _adj_ | B (SE B) | β | p | R^2^ _adj_ | B (SE B) | β | p | R^2^ _adj_ | B (SE B) | β | p | R^2^ _adj_ |
| ***Diagnostic cohort*** (N=104) |  |  |  |  |  |  |  |  |  |  |  |  |  |  |  |  |
|  |  |  |  |  |  |  |  |  |  |  |  |  |  |  |  |  |
| Comorbidity model |  |  |  | .09 |  |  |  | .07 |  |  |  | .15 |  |  |  | .19 |
| ADHD (diagnostic) | -.28 (.19) | -.15 | .159 |  | -.53 (.20) | -.30 | **.008** |  | -.72 (.20) | -.38 | **<.001** |  | -.94 (.20) | -.49 | **<.001** |  |
| Sex (female) | -.21 (.19) | -.11 | .272 |  | -.29 (.19) | -.16 | .132 |  | -.18 (.19) | -.09 | .364 |  | -.25 (.19) | -.13 | .183 |  |
| Age | -.05 (.04) | -.12 | .251 |  | .01 (.04) | .02 | .868 |  | -.03 (.04) | -.06 | .529 |  | .06 (.04) | .15 | .128 |  |
| Educational level | -.15 (.22) | -.07 | .508 |  | -.02 (.22) | -.01 | .939 |  | -.20 (.23) | -.09 | .377 |  | -.26 (.22) | -.11 | .242 |  |
| Anxiety conditions | -.68 (.33) | -.23 | .039 |  | -.03 (.33) | -.01 | .923 |  | -.28 (.34) | -.09 | .412 |  | .44 (.33) | .14 | .189 |  |
| Depression | .33 (.54) | .06 | .550 |  | .08 (.55) | .02 | .890 |  | 1.39 (.56) | .25 | .015 |  | -.12 (.55) | -.02 | .830 |  |
|  |  |  |  |  |  |  |  |  |  |  |  |  |  |  |  |  |
| Medication model |  |  |  | .06 |  |  |  | .12 |  |  |  | .11 |  |  |  | .19 |
| ADHD (diagnostic) | -.29 (.21) | -.16 | .170 |  | -.33 (.21) | -.18 | .112 |  | -.61 (.22) | -.32 | .**007** |  | -.80 (.21) | -.41 | **<.001** |  |
| Sex (female) | -.28 (.19) | -.15 | .137 |  | -.29 (.18) | -.16 | .113 |  | -.22 (.19) | -.11 | .265 |  | -.21 (.19) | -.10 | .271 |  |
| Age | -.05 (.04) | -.13 | .223 |  | .02 (.04) | .04 | .701 |  | .01 (.04) | .02 | .866 |  | .07 (.04) | .17 | .084 |  |
| Educational level | -.21 (.22) | -.10 | .341 |  | -.03 (.21) | -.02 | .877 |  | -.24 (.23) | -.10 | .305 |  | -.23 (.22) | -.10 | .305 |  |
| Medication | -.22 (.24) | -.11 | .361 |  | -.49 (.23) | -.23 | .037 |  | -.19 (.25) | -.09 | .445 |  | -.15 (.24) | -.07 | .526 |  |
| *Bold*: Significant result after Benjamini-Hochberg correction. *Note:* ADHD: attention-deficit/hyperactivity disorder; sex = assigned sex at birth. A higher score of reactivity indicates more intensity/frequency of the emotion, whereas a higher score of regulation indicates a better ability to regulate one’s emotions. Anxiety conditions include caregiver-reported diagnoses of unspecified anxiety, social anxiety disorder, generalized anxiety disorder and post-traumatic stress disorder. Ten participants with ADHD had co-occurring anxiety conditions and three participants with ADHD had co-occurring depression. A total of 23 participants with ADHD had ADHD medication (91.3% stimulants). Note that two participants with ADHD and five typically developing individuals had missing data on caregiver-reported diagnoses and medication, resulting in a reduced sample size for these variables (n=97) using pairwise deletion. | | | | | | | | | | | | | | | | |

**Table S6**. Post-hoc analyses of associations between diagnostic- and trait-level ADHD and caregiver-rated emotion-specific reactivity, while adjusting for co-occurring psychiatric symptoms and medication, respectively

|  | Sadness reactivity | | | | Fear reactivity | | | | Anger reactivity | | | | Exuberance reactivity | | | |
| --- | --- | --- | --- | --- | --- | --- | --- | --- | --- | --- | --- | --- | --- | --- | --- | --- |
|  | B (SE B) | β | p | R^2^ _adj_ | B (SE B) | β | p | R^2^ _adj_ | B (SE B) | β | p | R^2^ _adj_ | B (SE B) | β | p | R^2^ _adj_ |
| ***Diagnostic cohort*** (N=103) |  |  |  |  |  |  |  |  |  |  |  |  |  |  |  |  |
|  |  |  |  |  |  |  |  |  |  |  |  |  |  |  |  |  |
| Comorbidity model |  |  |  | .35 |  |  |  | .25 |  |  |  | .27 |  |  |  | -.00 |
| ADHD (diagnostic) | 1.11 (.19) | .53 | **<.001** |  | .79 (.21) | .36 | **<.001** |  | .86 (.23) | .36 | **<.001** |  | .37 (.20) | .21 | .069 |  |
| Sex (female) | .28 (.18) | .13 | .116 |  | .35 (.20) | .16 | .084 |  | .36 (.21) | .15 | .098 |  | -.06 (.19) | -.04 | .735 |  |
| Age | -.08 (.04) | -.17 | .040 |  | -.07 (.04) | -.14 | .135 |  | -.10 (.05) | -.19 | .030 |  | -.03 (.04) | -.06 | .553 |  |
| Educational level | .12 (.22) | .05 | .579 |  | .19 (.25) | .07 | .442 |  | -.25 (.26) | -.08 | .350 |  | -.11 (.23) | -.05 | .641 |  |
| Anxiety conditions | .42 (.32) | .12 | .193 |  | .92 (.37) | .24 | .014 |  | .56 (.39) | .14 | .153 |  | -.23 (.34) | -.07 | .513 |  |
| Depression | -.73 (.54) | -.12 | .182 |  | -.08 (.61) | -.01 | .900 |  | -1.73 (.65) | -.24 | **.009** |  | .16 (.57) | .03 | .777 |  |
|  |  |  |  |  |  |  |  |  |  |  |  |  |  |  |  |  |
| Medication model |  |  |  | .34 |  |  |  | .21 |  |  |  | .23 |  |  |  | .01 |
| ADHD (diagnostic) | 1.19 (.21) | .56 | **<.001** |  | .91 (.24) | .41 | **<.001** |  | .69 (.26) | .29 | **.009** |  | .41 (.22) | .23 | .068 |  |
| Sex (female) | .33 (.18) | .16 | .063 |  | .44 (.20) | .20 | .034 |  | .41 (.22) | .17 | .058 |  | -.08 (.19) | -.05 | .656 |  |
| Age | -.08 (.04) | -.18 | .034 |  | -.05 (.04) | -.11 | .225 |  | -.13 (.05) | -.24 | **.009** |  | -.02 (.04) | -.06 | .570 |  |
| Educational level | .16 (.22) | .06 | .464 |  | .26 (.25) | .10 | .296 |  | -.19 (.27) | -.07 | .467 |  | -.13 (.23) | -.06 | .582 |  |
| Medication | -.09 (.24) | -.04 | .702 |  | .12 (.27) | .05 | .666 |  | .39 (.29) | .14 | .179 |  | -.15 (.25) | -.07 | .534 |  |
| *Bold*: Significant result after Benjamini-Hochberg correction. *Note:* ADHD: attention-deficit/hyperactivity disorder; sex = assigned sex at birth. A higher score of reactivity indicates more intensity/frequency of the emotion, whereas a higher score of regulation indicates a better ability to regulate one’s emotions. Anxiety conditions include caregiver-reported diagnoses of unspecified anxiety, social anxiety disorder, generalized anxiety disorder and post-traumatic stress disorder. Ten participants with ADHD had co-occurring anxiety conditions and three participants with ADHD had co-occurring depression. A total of 25 participants with ADHD had ADHD medication (92.0 % stimulants). | | | | | | | | | | | | | | | | |

**Table S7**. Post-hoc analyses of associations between diagnostic- and trait-level ADHD and caregiver-rated emotion-specific regulation when adjusting for co-occurring psychiatric symptoms and medication, respectively

|  | Sadness regulation | | | | Fear regulation | | | | Anger regulation | | | | Exuberance regulation | | | |
| --- | --- | --- | --- | --- | --- | --- | --- | --- | --- | --- | --- | --- | --- | --- | --- | --- |
|  | B (SE B) | β | p | R^2^ _adj_ | B (SE B) | β | p | R^2^ _adj_ | B (SE B) | β | p | R^2^ _adj_ | B (SE B) | β | p | R^2^ _adj_ |
| ***Diagnostic cohort*** (N=103) |  |  |  |  |  |  |  |  |  |  |  |  |  |  |  |  |
|  |  |  |  |  |  |  |  |  |  |  |  |  |  |  |  |  |
| Comorbidity model |  |  |  | .22 |  |  |  | .24 |  |  |  | .23 |  |  |  | .17 |
| ADHD (diagnostic) | -.75 (.15) | -.49 | **<.001** |  | -.79 (.16) | -.47 | **<.001** |  | -.90 (.19) | -.48 | **<.001** |  | -.77 (.19) | -.41 | **<.001** |  |
| Sex (female) | -.08 (.14) | -.05 | .592 |  | -.08 (.15) | -.04 | .628 |  | -.07 (.17) | -.04 | .670 |  | -.08 (.18) | -.05 | .641 |  |
| Age | .01 (.03) | .03 | .770 |  | .04 (.03) | .10 | .257 |  | .02 (.04) | .04 | .662 |  | .03 (.04) | .08 | .423 |  |
| Educational level | -.05 (.17) | -.02 | .792 |  | -.07 (.19) | -.03 | .724 |  | .18 (.21) | .08 | .401 |  | .02 (.22) | .01 | .913 |  |
| Anxiety conditions | .03 (.26) | .01 | .910 |  | -.41 (.28) | -.14 | .147 |  | -.12 (.31) | -.04 | .705 |  | -.31 (.33) | -.10 | .345 |  |
| Depression | -.49 (.43) | -.11 | .259 |  | .29 (.47) | .06 | .532 |  | .24 (.53) | .04 | .647 |  | .73 (.55) | .13 | .184 |  |
|  |  |  |  |  |  |  |  |  |  |  |  |  |  |  |  |  |
| Medication model |  |  |  | .23 |  |  |  | .29 |  |  |  | .28 |  |  |  | .16 |
| ADHD (diagnostic) | -.67 (.17) | -.43 | **<.001** |  | -.62 (.18) | -.36 | **<.001** |  | -.67 (.20) | -.36 | **<.001** |  | -.72 | -.38 | **.001** |  |
| Sex (female) | -.06 (.14) | -.04 | .651 |  | -.10 (.15) | -.06 | .492 |  | -.07 (.17) | -.04 | .662 |  | -.12 | -.06 | .515 |  |
| Age | .01 (.03) | .02 | .826 |  | .05 (.03) | .12 | .158 |  | .03 (.04) | .07 | .439 |  | .04 | .10 | .309 |  |
| Educational level | -.04 (.17) | -.02 | .820 |  | -.10 (.18) | -.05 | .593 |  | .17 (.20) | .07 | .402 |  | .00 | -.00 | .986 |  |
| Medication | -.23 (.19) | -.13 | .227 |  | -.52 (.20) | -.26 | **.010** |  | -.52 (.22) | -.23 | .021 |  | -.14 | -.06 | .570 |  |
| *Bold*: Significant result after Benjamini-Hochberg correction. *Note:* ADHD: attention-deficit/hyperactivity disorder; sex = assigned sex at birth. A higher score of reactivity indicates more intensity/frequency of the emotion, whereas a higher score of regulation indicates a better ability to regulate one’s emotions. Anxiety conditions include caregiver-reported diagnoses of unspecified anxiety, social anxiety disorder, generalized anxiety disorder and post-traumatic stress disorder. Ten participants with ADHD had co-occurring anxiety conditions and three participants with ADHD had co-occurring depression. A total of 25 participants with ADHD had ADHD medication (92.0 % stimulants). | | | | | | | | | | | | | | | | |
